# Supplementary material for: The Interaction of the Senescent and Adjacent Breast Cancer Cells Promotes the Metastasis of Heterogeneous Breast Cancer Cells through Notch Signaling
Source: Int J Mol Sci. 2021 Jan 15;22(2):849. doi: 10.3390/ijms22020849 (PMC7830992; doi:10.3390/ijms22020849)
Supplement: Supplementary file 1 [file ijms-22-00849-s001.pdf]

Supplementary Information

The interaction of the senescent and adjacent breast cancer cells promotes the metastasis of heterogeneous breast cancer cells through Notch signaling

Zhang et al

Supplementary Figures

Figure S1. Doxorubicin induced cellular senescence in MCF-7 cells.

Figure S2. Generation of the co-culture model.

Figure S3. Detection of DAPT concentration.

Figure S4. SA-β-gal activity of the co-cultured T47D cells was significantly decreased in the co-culture system.

Figure S5. DAPT inhibited EMT progression and Notch signaling of breast cancer MCF-7-luc cells in the co-culture system.

Figure S6. The re-entry of senescent MCF-7 breast cancer cells into the cell cycle was independent of activation of Notch signaling after co-culture with adjacent breast cancer cells.

Figure S7. Changes in the Wnt signaling pathway in senescent breast cancer MCF-7 cells.

Supplementary Figure Legends

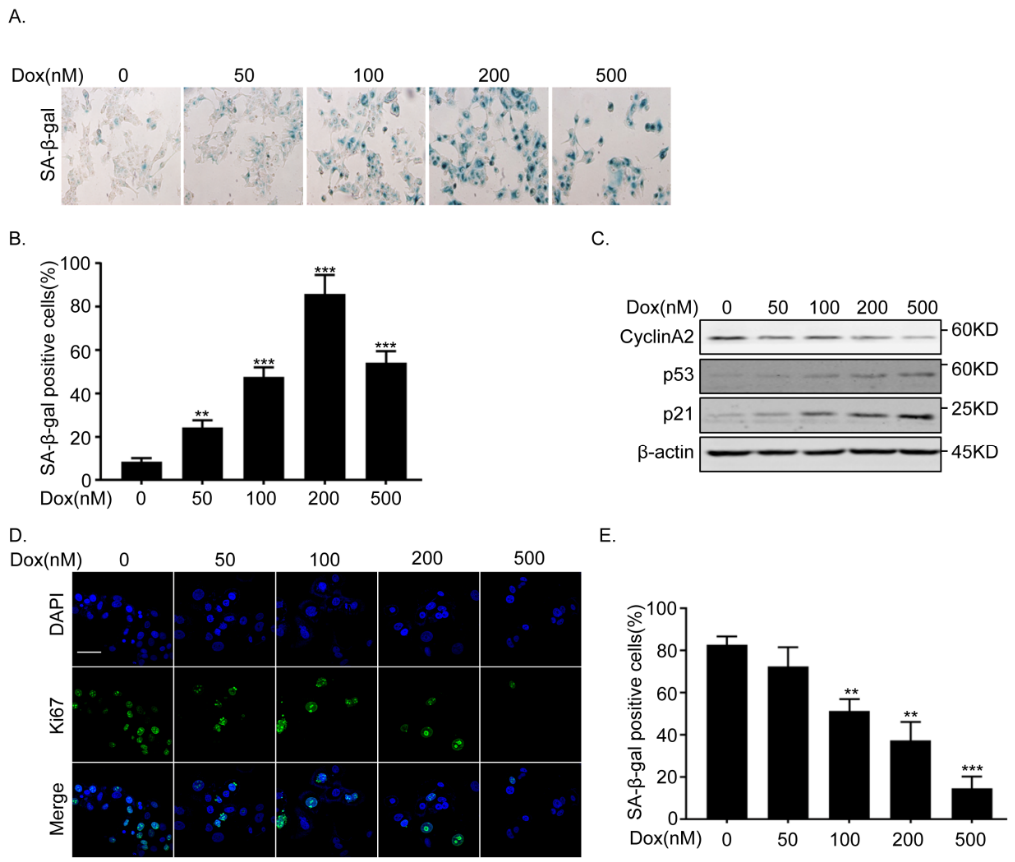

Figure S1. Doxorubicin induced cellular senescence in MCF-7 cells.

A,B.The β-Gal staining analysis of MCF-7 cells treated with different concentrations of doxorubicin (0, 50, 100, 200 and 500nM).(A)Representative images. (B) Percentage of β-gal-positive cells (Error bars indicate mean SD, n = 3 experimental replicates, $^{*}P< 0.05$, $^{**}P< 0.01$, $^{***}P < 0.001$).

C. Immunoblot analysis of protein expression of cylinA2, p53 and p21 in MCF-7 cells treated with different concentrations of doxorubicin (0, 50, 100, 200 and 500nM).

D, E. Immunofluorescence staining of the proliferation marker, Ki67, in MCF-7 cells treated with different concentrations of doxorubicin (0, 50, 100, 200 and 500nM).

Scale bar: 50μm

(D) Representative micrographs of Ki67-positive MCF-7 cells treated with different concentrations of doxorubicin (0, 50, 100, 200 and 500nM). (E) Data represent the number of cells derived from mean cell counts of three fields (Error bars indicate mean SD, n = 3 experimental replicates, $^{*}P< 0.05$, $^{**}P< 0.01$, $^{***}P < 0.001$).

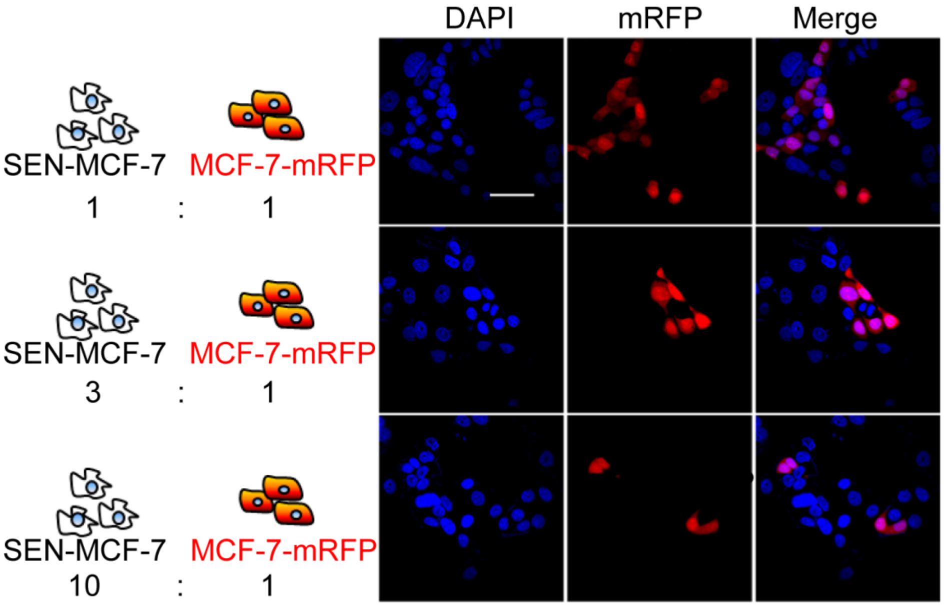

Figure S2. Generation of the co-culture model.

SEN-MCF-7 and MCF-7-mRFP cells were plated at ratios of 1: 1, 3: 1, and 10: 1, mixed and returned to the incubator for continuous culture for 3 days. The optimal co-culture ratio was determined. Scale bar: 50μm

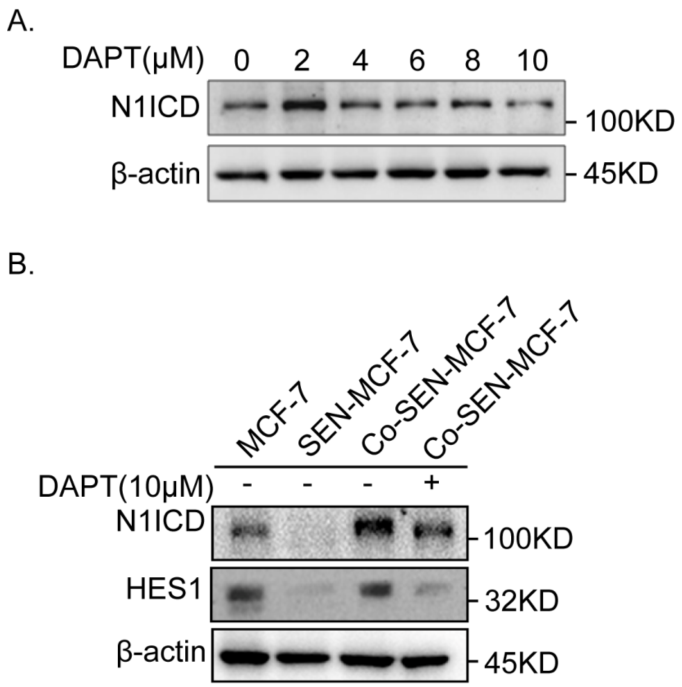

Figure S3.Detection of DAPT concentration.

A. Immunoblot analysis of protein expression of N1ICD treated with different concentrations of DAPT (0, 2, 4, 6, 8, 10μM) in MCF-7 cells.

B. Immunoblot analysis of protein expression of N1ICD and HES1 treated with DAPT (10μM) in MCF-7 cells.

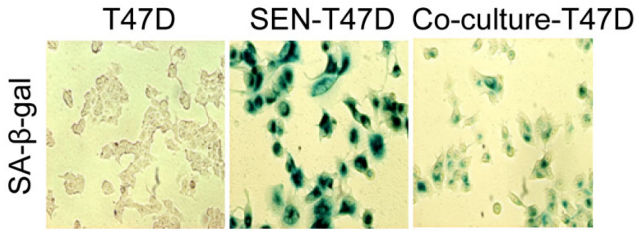

Figure S4. SA-β-gal activity of the co-cultured T47D cells was significantly decreased in the co-culture system.

β-Gal staining analysis of T47D cells subjected to the indicated treatments.

Representative images.

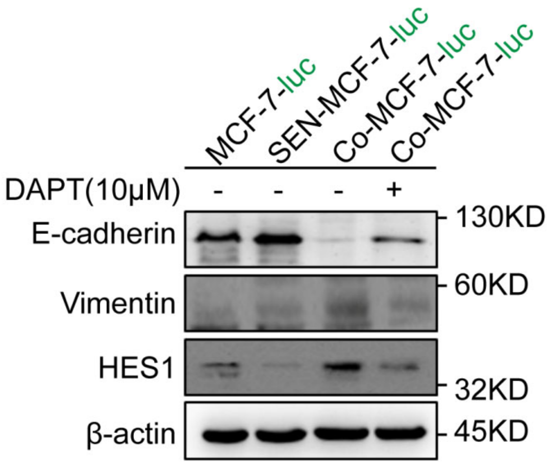

Figure S5. DAPT inhibited EMT progression and Notch signaling of breast cancer MCF-7-luc cells in the co-culture system

Immunoblot analysis of protein expression of the E-cadherin, Vimentin and Hes1 in MCF-7-luc., SEN-MCF-7-luc. and Co-culture-MCF-7-luc.cells with the indicated treatments.

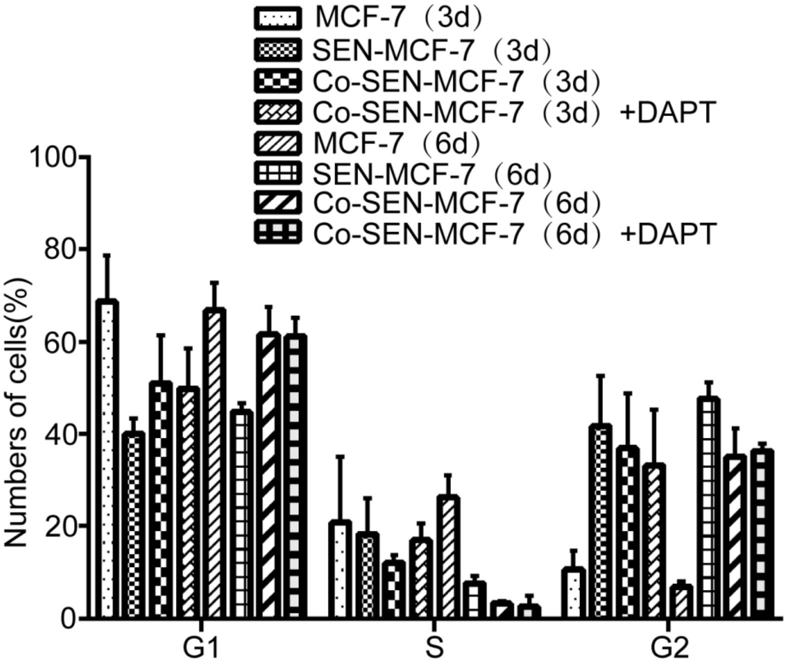

Figure S6.The re-entry of senescent MCF-7breast cancer cells into the cell cycle was independent of activation of Notch signaling after co-culture with adjacent breast cancer cells.

Cell cycle analysis of MCF-7, SEN-MCF-7 and Co-SEN-MCF-7 cells with the indicated treatments.

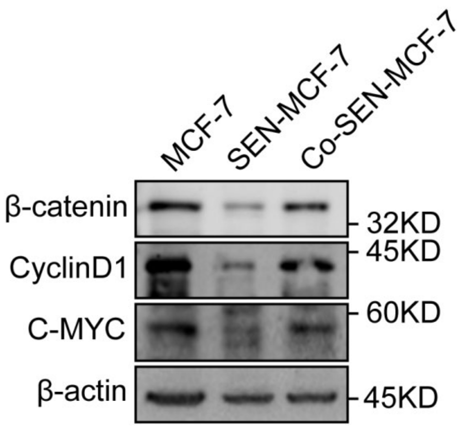

Figure S7.Changes in the Wnt signaling pathway in senescent breast cancer MCF-7 cells.

Immunoblot analysis of protein expression of β-catenin and the canonical Wnt-targets, cyclinD1 and C-MYC, in MCF-7 cells.
